# A Case of Prolonged Recovery for Post-percutaneous Coronary Intervention (PCI) Sodium-Glucose Cotransporter-2 (SGLT2) Inhibitor-Induced Euglycemic Diabetic Ketoacidosis in a 28-Year-Old

**DOI:** 10.7759/cureus.45180

**Published:** 2023-09-13

**Authors:** Cyrena Petersen, Frederick Gyabaah, Jose Sotelo, Sandeesh Yohanna, Abhizith Deoker

**Affiliations:** 1 Internal Medicine, Texas Tech University Health Sciences Center Paul L. Foster School of Medicine, El Paso, USA; 2 Internal Medicine, Texas Tech University Health Sciences Center, El Paso, USA

**Keywords:** drug-related side effects and adverse reactions, post pci, types 2 diabetes, sodium-glucose cotransporter-2 (sglt2) inhibitors, euglycaemic diabetic ketoacidosis

## Abstract

Euglycemic diabetic ketoacidosis (DKA) is a rare, but clinically important, presentation that can lead to significant morbidity and mortality in patients with diabetes mellitus. It has been associated with multiple etiologies, including sodium-glucose cotransport-2 (SGLT2) inhibitor use. This case report details the presentation of a 28-year-old male patient who was recently diagnosed with non-ST elevated myocardial infarction (NSTEMI) status post-percutaneous coronary intervention (PCI) to left anterior descending (LAD) and type 2 diabetes mellitus (T2DM) and discharged on a new medical regiment that included an SGLT2 inhibitor. The patient presented five days later with dyspnea, nausea, and vomiting. On initial evaluation, he had tachycardia and hypertension. Lab work revealed hyperkalemia, metabolic anion gap acidosis, and the presence of ketones and glucose in the urine, which led to the diagnosis of euglycemic DKA. The patient was started on intravenous (IV) insulin, bicarbonate, and D5 ½ normal saline (NS) and required five days of continuous treatment for the anion gap to close.

Considering studies have shown that SGLT2 inhibitors are associated with euglycemic DKA, it is proposed that the use of an SGLT2 inhibitor in this newly diagnosed, post-PCI patient led to the development of euglycemic DKA. DKA most commonly resolves within 24 hours of treatment; however, our patient did not recover until after 120 hours of treatment. Recent studies have suggested that SGLT2-inhibitor euglycemic DKA may be associated with longer recovery time; however, there is still a need to further research the consistency of these findings and quantify the estimated duration of treatment across populations. There is also a need for investigation into how co-morbid factors, such as a recent NSTEMI and PCI, may affect recovery times or predispose patients who are taking SGLT2-inhibitors to develop euglycemic DKA as SGLT2 inhibitors are being more widely prescribed. This case report highlights the importance of creating more detailed and evidence-based guidelines for prescribing SGLT2 inhibitors for patients with diabetes and encourages more research into the expected duration of treatment for patients with SGLT2-induced euglycemic DKA and factors that may affect it.

## Introduction

Euglycemic diabetic ketoacidosis (DKA) is characterized by metabolic acidosis with a pH less than 7.3 and serum bicarbonate less than 18 mEq/L, ketosis, and blood glucose levels less than 200 mg/dL [1]. It has been associated with sodium-glucose cotransporter-2 (SGLT2) inhibitor use, pregnancy, lower calorie intake, alcohol use, insulin prior to admission, cocaine use, pancreatitis, sepsis, chronic liver disease, and liver cirrhosis [2]. In recent years, there has been an increase in SGLT2 inhibitor use and with it an increase in associated euglycemic DKA incidence [2-4].

SGLT2 inhibitors are hypoglycemic agents that reduce glucose in the blood by antagonizing SGLT2 receptors in the proximal renal tubules, which prevents reabsorption and increases the excretion of glucose [5]. The drug has been used for patients with diabetes mellitus and has been associated with additional health outcomes such as improvements in weight loss, dyslipidemia, non-alcoholic fatty liver disease, and insulin resistance. Recent studies have shown that SGLT2 inhibitors can also be helpful in the management of congestive heart failure symptoms [6].

Euglycemic DKA remains a rare complication of SGLT2 inhibitor use; however, it is a life-threatening complication. This case report details the management of a 28-year-old male who was status post-percutaneous coronary intervention and recently diagnosed with type two diabetes mellitus, who underwent a prolonged recovery time for SGLT2 inhibitor-induced euglycemic diabetic ketoacidosis. It seeks to contribute to the ongoing literature on the association between SGLT2 inhibitor use and euglycemic DKA to lead to better medical management of SGLT2 inhibitors in patients with diabetes.

## Case presentation

**Figure 1:**
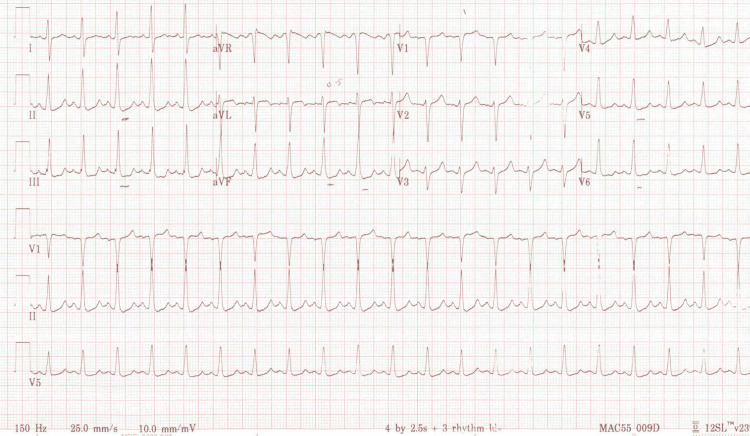
EKG showing sinus tachycardia with ST depression in leads II, III, and aVF from the patient’s initial presentation

A 28-year-old male with no past medical history originally presented to the emergency department (ED) for chest pain and was diagnosed with non-ST elevated myocardial infarction (NSTEMI) status post-percutaneous coronary intervention (PCI) to left anterior descending (LAD) and type 2 diabetes mellitus (T2DM). Upon discharge, the patient was started on aspirin 81 mg daily, insulin glargine 14 units daily, insulin aspart two units daily, metoprolol 50 mg twice daily (BID), rosuvastatin 40 mg daily, ticagrelor 90 mg BID, pantoprazole 40 mg every two days, and empagliflozin 10 mg daily. The patient presented to the ED again five days after his previous discharge with dyspnea, nausea, vomiting, food intolerance, chills, dizziness, lightheadedness, and constipation for one day. He reported compliance with medication since discharge. His vitals were significant for tachycardia with a heart rate of 123 beats per minute and hypertension with a blood pressure of 173/87 mmHg. The complete blood count (CBC) results showed leukocytosis with neutrophilia, elevated hemoglobin, and elevated hematocrit. The comprehensive metabolic panel showed hyperkalemia, undetectable bicarbonate level, mild hyperglycemia, and elevated alanine transaminase (ALT). The anion gap was undeterminable because the bicarbonate level was undetectable. Venous blood gas (VBG) showed severe acidemia, hypocapnia, hypobicarbonatemia, and hyperlactatemia. The urinalysis showed glucosuria and ketonuria. The hemoglobin A1C was severely elevated. The beta-hydroxybutyrate level was elevated (Table 1). An electrocardiogram (EKG) showed sinus tachycardia with ST depression in leads II, III, and aVF (Figure 1).

**Table 1:** Patient’s initial lab values

Labs	Patient’s Lab Values	Reference Ranges
Complete Blood Count		
White Blood Cell Count	16,100 /uL	4,500-11,000 /uL
Absolute Neutrophil Count	12,850 /uL	2,500-6,000 /uL
Hemoglobin	18.4 g/dL	13.5-17.5 g/dL
Hematocrit	56.4 %	41 – 53 %
Complete Metabolic Panel		
Sodium	135 mmol/L	135-145 mmol/L
Potassium	5.8 mmol/L	3.5 – 5.5 mmol/L
Chloride	101 mmol/L	96 – 106 mmol/L
Bicarbonate	< 5 mEq/L	23 – 29 mmol/L
Glucose	154 mg/dL	< 100 mg/dL
Anion Gap	Undetectable	8 - 12 mmol/L
Aspartate Transaminase	44 U/L	8 – 33 U/L
Alanine Transaminase	81 U/L	7 – 56 U/L
Venous Blood Gas		
pH	7.048	7.31 – 7.41
pCO2	25.5 mmHg	35 – 45 mmHg
Bicarbonate	7.0 mEq/L	23 – 29 mmol/L
Lactate	2.1 mmol/L	< 1.0 mmol/L
Urinalysis		
Glucose	500 mg/dL	0 – 15 mg/dL
Ketones	> 80 mg/dL	< 1 mg/dL
Other		
Hemoglobin A1C	11.1 %	4.0 – 5.6 %
Beta-hydroxybutyrate	9.00 mmol/dL	< 0.6 mmol/L

While in the ED, the patient was started on 1 L of normal saline (NS). Laboratory analysis showed the patient to be in euglycemic diabetic ketoacidosis (DKA) likely secondary to the use of an SGLT2 inhibitor and the medical intensive care unit (MICU) was consulted. The patient was given a sodium bicarbonate IV push and started on an insulin drip, bicarbonate drip, and D5 ½ NS. The patient's care was then transferred to the MICU. The home medications of aspirin, ticagrelor, and rosuvastatin were continued. Empagliflozin was held. Enoxaparin 40 mg was added for deep vein thrombosis (DVT) prophylaxis. The insulin drip was continued for the next three days. By the fifth day, the anion gap had returned to baseline and the bicarbonate had increased to near normal range (Table 2). Based on this improvement, the patient was transferred to the general medical floor, taken off the insulin and bicarbonate drips, and started on insulin glargine 50 units daily. Elevated aminotransferases were noted at this time. This increase was most likely due to the use of rosuvastatin; therefore, the dose was decreased from 40 mg to 20 mg daily. The patient was counseled to remain in the hospital for further monitoring of liver enzymes; however, the patient chose to follow up with monitoring of his liver enzymes outpatient and was discharged.

**Table 2:** Patient’s hospital day 5 lab values

Labs	Patient’s Lab Values	Reference Ranges
Complete Blood Count		
White Blood Cell Count	5.85 /uL	4,500-11,000 /uL
Absolute Neutrophil Count	3.24 /uL	2,500-6,000 /uL
Hemoglobin	15.6 g/dL	13.5-17.5 g/dL
Hematocrit	43.8 %	41 – 53 %
Complete Metabolic Panel		
Sodium	133 mmol/L	135-145 mmol/L
Potassium	3.8 mmol/L	3.5 – 5.5 mmol/L
Chloride	21 mmol/L	96 – 106 mmol/L
Bicarbonate	21 mEq/L	23 – 29 mmol/L
Glucose	154 mg/dL	< 100 mg/dL
Anion Gap	10 mmol/L	8 - 12 mmol/L
Aspartate Transaminase	222 U/L	8 – 33 U/L
Alanine Transaminase	368 U/L	7 – 56 U/L

## Discussion

The mechanism of action for the development of euglycemic DKA in patients who use SGLT2 inhibitors is still unknown; however, recent studies have proposed different mechanisms. Ogawa et al. suggest that the reduced glucose and insulin levels that result from SGLT2 inhibitor use lead to increased lipolysis, free fatty acid production, and beta-oxidation of the free fatty acids [7]. The increased beta-oxidation results in increased ketone body production and the development of euglycemic DKA.

The incidence of euglycemic DKA secondary to SGLT2 inhibitor use is low. However, there are precipitating factors that can predispose patients to this complication. Recent studies have found that infection, severe acute illness, cerebrovascular events, dehydration, ketogenic diet, and surgery, amongst other factors, can increase the likelihood of DKA in patients who use SGLT2 inhibitors [8-15]. Several of these factors were present for our patient and possibly contributed to the development of euglycemic DKA.

A systematic review and quantitative analysis by Dutta et al. found that the most common predisposing factors for euglycemic DKA for SGLT2 inhibitor users were female gender, recent surgery, and canagliflozin, with concomitant metformin use [16]. Our patient was male and using empagliflozin, which differs from the previously described typical patient. Our patient was also 28 years old with an initial diagnosis of T2DM only one week prior and had been taking empagliflozin for only eight days prior to being diagnosed with euglycemic DKA.

Previous studies have found an association between the development of euglycemic DKA and recent surgery in patients who are on SGLT2 inhibitors. There is no current evidence indicating whether there are particular surgical procedures that have a higher risk of euglycemic DKA development; however, there have been some reports of euglycemic DKA in SGLT2 inhibitor users after cardiac procedures and bariatric surgery [16-17]. Some studies have recommended holding SGLT2 inhibitors for 72 hours prior to surgeries. Mehta et al. conducted a retrospective cohort study that found that SGLT2 inhibitor-induced euglycemic DKA was less likely to occur in patients undergoing non-emergent procedures when compared to patients undergoing emergent surgeries [18]. The authors attributed this difference to the ability to stop SGLT2 inhibitor use three days before the non-emergent procedures, which was not possible for the emergent procedures. In addition to holding SGLT2 inhibitors before surgeries, there have also been recommendations to counsel patients on “sick day rules” where patients are advised to hold their SGLT2 inhibitor for the duration of any acute illness [15].

The initial goals of treatment for patients with DKA include fluid resuscitation, correction of acidosis, and prevention of hypokalemia. Patients are usually given IV normal saline, IV insulin, and potassium if serum potassium is below 5.3 Eq/L [19]. The anion gap is the primary metric that is used to determine the resolution of DKA. Once treatment is initiated, the anion gap is usually corrected within 24 hours. Our patient experienced a prolonged recovery time, and the anion gap was not corrected until his fifth day on the treatment protocol, which is five times the normal recovery time. Recent studies have found that euglycemic DKA in patients who are using SGLT2 inhibitors has required a prolonged treatment regimen; however, there is still a need for further research on the consistency of these findings and the estimated duration of treatment across populations [19-20]. Since there is no current consensus in the literature on estimated recovery time for SGLT2 inhibitor-induced DKA, we are unable to ascertain whether our patient's recovery time was further prolonged by a confounding factor from his history. Also, while the standard DKA treatment protocol was followed for this patient, the prolonged recovery time brings into question whether there are alterations in the typical management of DKA for patients with SGLT2 inhibitor-induced DKA that might be considered in the future that would allow for a more efficient recovery time.

This case report highlights the need for further investigation into which surgeries have a higher association with euglycemic DKA in SGLT2 inhibitor users, whether the age of the patient or dosage of SGLT2 inhibitors has an association with euglycemic DKA in SGLT2 inhibitor users, and what is an appropriate window to hold SGLT2 inhibitors for those with an acute illness or recent surgical event. As SGLT2 inhibitor use continues to rise, it is important for clinicians to have an evidence-based understanding of what risk factors can predispose patients to complications, such as euglycemic DKA, and if there are alterations in the medication dosage, administration, or formula that can help prevent these complications. Also, there is a need for further research and clinical guidance on the management of SGLT2 inhibitor-induced DKA to determine an expected recovery time and if alterations from the standardized DKA management are more beneficial for patients with SGLT2 inhibitor-induced DKA.

## Conclusions

While euglycemic DKA is a rare presentation, its association with SGLT2 inhibitors needs to be further investigated. SGLT2 inhibitors are being more readily prescribed by physicians, as they have been shown to reduce blood sugar for patients with diabetes and decrease the risk of adverse cardiovascular events for patients with heart failure. Further characterizing risk factors for and effective management of euglycemic DKA in patients who use SGLT2 inhibitors can lead to better medical management and allow for safe and efficacious use of SGLT2 inhibitors for patients who have found this to be a clinically significant medication.
